# Clinical trio genome sequencing facilitates the interpretation of variants in cancer predisposition genes in paediatric tumour patients

**DOI:** 10.1038/s41431-023-01423-8

**Published:** 2023-07-28

**Authors:** Christopher Schroeder, Ulrike Faust, Luisa Krauße, Alexandra Liebmann, Michael Abele, German Demidov, Leon Schütz, Olga Kelemen, Alexandra Pohle, Silja Gauß, Marc Sturm, Cristiana Roggia, Monika Streiter, Rebecca Buchert, Sorin Armenau-Ebinger, Dominik Nann, Rudi Beschorner, Rupert Handgretinger, Martin Ebinger, Peter Lang, Ursula Holzer, Julia Skokowa, Stephan Ossowski, Tobias B. Haack, Ulrike A. Mau-Holzmann, Andreas Dufke, Olaf Riess, Ines B. Brecht

**Affiliations:** 1 Institute of Medical Genetics and Applied Genomics, University Hospital Tübingen, Tübingen, Germany; 2 Centre for Personalized Cancer Prevention, University Hospital Tübingen, Tübingen, Germany; 3 Department of Paediatric Haematology and Oncology, University Children’s Hospital Tübingen, Tübingen, Germany; 4 Department of Paediatric Haematology and Oncology, Children’s Hospital Heilbronn, Heilbronn, Germany; 5 Institute of Pathology and Neuropathology, University Hospital Tübingen, Tübingen, Germany; 6 Department of Oncology, Haematology, Immunology, Rheumatology, and Pulmonology, University Hospital Tübingen, Tübingen, Germany; 7 NGS Core Centre Tübingen, University Tübingen, Tübingen, Germany

**Keywords:** Risk factors, Genetics, Health care

## Abstract

The prevalence of pathogenic and likely pathogenic (P/LP) variants in genes associated with cancer predisposition syndromes (CPS) is estimated to be 8-18% for paediatric cancer patients. In more than half of the carriers, the family history is unsuspicious for CPS. Therefore, broad genetic testing could identify germline predisposition in additional children with cancer resulting in important implications for themselves and their families. We thus evaluated clinical trio genome sequencing (TGS) in a cohort of 72 paediatric patients with solid cancers other than retinoblastoma or CNS-tumours. The most prevalent cancer types were sarcoma (*n* = 26), neuroblastoma (*n* = 15), and nephroblastoma (*n* = 10). Overall, P/LP variants in CPS genes were identified in 18.1% of patients (13/72) and P/LP variants in autosomal-dominant CPS genes in 9.7% (7/72). Genetic evaluation would have been recommended for the majority of patients with P/LP variants according to the Jongmans criteria. Four patients (5.6%, 4/72) carried P/LP variants in autosomal-dominant genes known to be associated with their tumour type. With the immediate information on variant inheritance, TGS facilitated the identification of a de novo P/LP in *NF1*, a gonadosomatic mosaic in *WT1* and two pathogenic variants in one patient (*DICER1 and PALB2*). TGS allows a more detailed characterization of structural variants with base-pair resolution of breakpoints which can be relevant for the interpretation of copy number variants. Altogether, TGS allows comprehensive identification of children with a CPS and supports the individualised clinical management of index patients and high-risk relatives.

## Introduction

Cancer in children is a rare event and differs substantially in pathogenetic origin, type and frequency of neoplasms as well as associated genetic alterations from cancer in adulthood. It is generally believed that the occurrence of sporadic paediatric malignancies is based on a developmental error that occurs during embryogenesis either due to genetic or environmental factors, while cancer predisposition syndromes (CPS) were considered rare [1]. The hypothesis of a link between disturbed developmental processes and oncogenesis is supported by well-established genetic disorders with dysmorphic features and increased cancer risk, like Beckwith-Wiedemann syndrome, WAGR-syndrome or Down syndrome [1, 2].

Based on these and other specific findings on the aetiology of childhood malignancies, a set of criteria ("Jongmans Criteria") has been developed for clinicians to help identify patients who might benefit from referral to a clinical geneticist [3]. Those criteria include, besides family history, the occurrence of second malignancies, specific tumour entities with a high rate of cancer predisposition (e.g., adrenocortical carcinoma for *TP53*), excessive toxicity and other specific features as well as paediatric patients with rare or adult-type tumours [2, 3]. However, a non-negligible fraction of families seems to go undetected using clinical criteria, due to de-novo variants, reduced penetrance, or variable manifestation of cancer genes among other factors [4–6]. Thus, discussions are ongoing if comprehensive genetic testing should be applied as a first-tier test in diagnostics.

Broad sequencing studies in the paediatric cancer population have revealed an unexpectedly high rate (8–18%) of pathogenic and likely pathogenic (P/LP) germline variants in CPS genes depending on tumour type and disease stage [5, 7–12]. The interpretation of the results remains challenging since not all genes are known to be associated with paediatric cancer or the patient’s tumour entity. Still, the detection of an underlying P/LP germline variant is important for patients as it may help to reduce morbidity and mortality rates for patients and their family members. Consequences may include treatment plan modifications, enrolment in surveillance programs, and/or predictive testing of family members. The positive impact on survival of such measures was shown by applying an extensive surveillance protocol for Li-Fraumeni syndrome patients [13, 14].

The current standard for diagnostic cancer predisposition testing is targeted panel sequencing or exome sequencing focused on aberrations in known CPS genes. Genome sequencing is being adopted in an increasing number of laboratories and has several advantages over the two aforementioned approaches: (1) improved detection of structural variants (e.g. inversions, translocations, CNVs) including break points with base pair resolution; (2) detection of variants in regulatory regions as well as deep intronic variants; and (3) detection of repeat expansions [15–21]. These advantages have been translated into higher detection rates of clinically significant findings in different disease areas [22, 23]. Trio parent-child Genome Sequencing (TGS) provides additional information with regard to allelic distribution, mode of inheritance, and a potentially higher sensitivity for genetic variants due to internal error correction [24].

With this study, we evaluated the utility of clinical TGS in a cohort of paediatric cancer patients and its feasibility in routine clinical practice. Of special interest was the added value regarding allele information and increased sensitivity for structural variants.

## Material and methods

### Study cohort

**Fig. 1:**
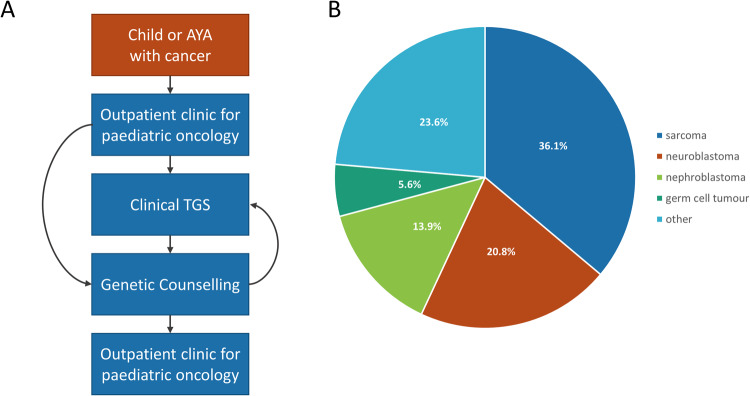
Summary of the study approach and patient cohorts. **A** Study protocol and patient path used in this study. TGS Trio parent-child Genome Sequencing. **B** Overview of cancer types investigated in this study.

Patients were recruited from the Department of Paediatric Haematology and Oncology at the University Children’s Hospital Tübingen. All patients treated for malignant solid tumours (excluding central nervous system (CNS) tumours and retinoblastoma) within the department between 01/2019 and 06/2020 were offered genetic testing in a personal educational interview (Fig. 1A). Furthermore, all follow-up patients in our department with a solid (non-CNS) tumour diagnosis between 01/2000 and 12/2018, were invited by letter and/or personal communication during routine follow-up. All patients were seen by a clinician or clinical geneticist before and/or after genetic testing at our interdisciplinary outpatient clinic for hereditary childhood cancer (Cooperative Clinic of the Institute of Medical Genetics and Applied Genomics and the Department of Paediatric Haematology and Oncology, University Hospital Tübingen) of the Centre for Rare Diseases Tübingen between 01/2019 and 08/2020. Informed consent was obtained from children and their parents (project ID of the local ethics committee 367/2019BO1 and 819/2017BO1, Clinical trial registration number: NCT03954652). During the appointment, the family history and clinical data were recorded. While patients and families were invited for genetic testing independently of a specific indication for diagnostic screening for cancer disposition, the indication for germline testing was evaluated retrospectively by using a questionnaire developed by Jongmans et al. (2016) and modified by the cancer predisposition syndrome (CPS) working group of the German Society for Paediatric Oncology and Haematology (GPOH) [2, 3]. Genetic counselling was offered to all patients included in this study. All patients with genetic findings in CPS genes were provided with psychological support and invited to participate in cancer screening in the specialized cancer outpatient clinic for hereditary childhood cancer.

### Genome sequencing and data analysis

Genome sequencing was performed on genomic DNA from blood as previously described [25]. In brief, libraries were prepared using an Illumina TruSeq PCR-free kit (Illumina, San Diego, USA) and subsequently sequenced on an Illumina NovaSeq6000 instrument (Illumina, San Diego, USA) at 151 bp paired-end reads. Sequences were analysed using the megSAP pipeline (https://github.com/imgag/megSAP/tree/GRCh37). BWA-mem was used for mapping against GRCh37, and alignments were post-processed by various tools (e.g. indel realignment with ABRA2 and PCR duplicate marking with SAMBLASTER) [26–28]. Germline variants were detected using freebayes for single nucleotide variants and indels (https://github.com/freebayes/freebayes), ClinCNV (https://www.tdx.cat/handle/10803/668208) for germline copy-number variants, and Manta for other types of structural variants [29]. A combination of tools and databases, e.g., Ensembl’s VEP, COSMIC (https://cancer.sanger.ac.uk/cosmic) and in-house data from previously sequenced patients, was used for annotation of variants [30]. The resulting variant lists were inspected and further refined using the decision support system *GSvar* (https://github.com/imgag/ngs-bits/blob/master/doc/GSvar/index.md), which includes various filters and visualisation options. Variants were filtered according to their predicted impact (VEP HIGH and MODERATE) as well as minor allele frequency in public databases and an in-house database containing >20,000 datasets from individuals with unrelated phenotypes (MAF ≤ 0.1% for AD genes and ≤ 1% for AR genes). Furthermore, the variants were reduced to a target region that included the most important CPS genes (Supplementary Table 1). Copy number variants (CNVs) were filtered by log-likelihood ( ≥ 12 scaled by regions) and occurrence in an in-house database. Selected variants were visually validated with the Integrative Genomics Viewer (http://software.broadinstitute.org/software/igv/) and classified in accordance with the American College of Medical Genetics and Genomics (ACMG) guidelines [31]. Variants of unknown clinical significance (VUS) and P/LP variants in phenotype-associated CPS genes were reported based on OMIM for index patients. In the event of a secondary finding within an extended set of the ACMG genes, only P/LP variants were reported for the index patient and their parents depending on actionability. Results were compared to the non-cancer group in gnomAD v3.1.2 (gnomad.broadinstitute.org) [32]. To identify variants in novel candidate genes, we searched for de novo variants. Additional tests, e.g., diagnostic karyotyping or diagnostic tumour sequencing, were added in selected cases to confirm the molecular diagnosis or support variant classification.

## Results

In total, 72 childhood tumour patients and their parents were included in this study. The male:female ratio was 1.18 (male *n* = 39, female *n* = 33) and the median age of first tumour detection was 4.75 years (range 0–20.5 years). Additional characteristics of the patients can be found in the Supplementary Table 2. The most prevalent tumour entities were sarcoma (36.1%, *n* = 26), neuroblastoma (20.8%, *n* = 15), and nephroblastoma (13.9%, *n* = 10) (Fig. 1B). Within the heterogeneous group of sarcomas, the most common types were rhabdomyosarcoma (*n* = 12), Ewing sarcoma (*n* = 7), and osteosarcoma (*n* = 3). Additional information on patients, their families and genetic findings can be found in Table 1. TGS identified P/LP variants in CPS genes in 18.1% (13/72) of patients including dominant and recessive CPS genes. Most variants were small nucleotide variants or indels and four patients were found to carry structural variants in CPS genes, i.e. three patients with larger deletions and one patient with a duplication.

**Table 1:** Patients with pathogenic variants, likely pathogenic variants and variants of unknown significance.

Trio ID	Sex	Tumours	AAO years	Gene	Geno-type	Variant	Class	Inheritance
PaedCan01	m	Nephroblastomatosis	0.6	*WT1* (AD)	het	ENST00000332351:c.1046del:p.(Leu349Profs*26)	P	gonadosomatic mosaicism
PaedCan02	m	Neuroblastoma	0.2	*MITF* (AR, AD)	het	ENST00000394351:c.952G>A:p.(Glu318Lys)	LP	maternal
PaedCan07	m	Embryonal rhabdomyosarcoma	1.2	*BUB1B* (AR)	het	ENST00000412359:c.2577+5G>A:p.(?)	LP	maternal
				*BUB1B* (AR)	het	ENST00000412359:c.2405_2406del:p.(Ser802Cysfs*29)	LP	paternal
PaedCan08	f	Non-Hodgkin Lymphoma	2.6	*ATM* (AR, AD)	het	ENST00000278616:c.1516G>T:p.(Gly506Cys)	VUS	paternal
		Basal cell carcinoma	17.4	*PALB2* (AD)	het	ENST00000261584:c.1843C>G:p.(Pro615Ala)	VUS	paternal
		Urothelial carcinoma	25.0					
		Breast cancer	27.2					
PaedCan09	f	Neuroblastoma	3.0	*RECQL4* (AR)	het	ENST00000428558:c.1573del:p.(Cys525Alafs*33)	P	maternal
		Fibroma of the ovary	20.2	*SEC23B*	het	ENST00000262544.6:c.2101C>T:p.(Arg701Cys)	VUS	maternal
		Basal cell carcinoma	20.8					
		Melanocanthoma	21					
		Thyroid adenoma	21					
		FNH of the liver	22					
PaedCan15	m	MELTUMP	9.7	*MUTYH* (AR)	het	ENST00000450313.1:c.1187G>A:p.(Gly396Asp)	P	maternal
PaedCan16	f	Papillary thyroid cancer	15.7	*DICER1* (AD)	het	chr14:95592950-96702914del [includes DICER1 (ENST00000343455) Ex1-7]	P	maternal
				*PALB2* (AD)	het	ENST00000261584:c.72del:p.(Arg26Glyfs*7)	P	maternal
PaedCan18	m	Low grade malignant Mesenchymal tumour, NOS	12.1	*NF1* (AD)	het	chr17:29563720-29849063del [includes NF1 (ENST00000356175) Ex30-58]	P	de novo
PaedCan32	f	Inflammatory myofibroblastic tumour	2.6	*MUTYH* (AR)	het	ENST00000450313.1:c.1187G>A:p.(Gly396Asp)	P	paternal
PaedCan39	f	Embryonal rhabdomyosarcoma	6.8	*NF1* (AD)	het	ENST00000356175:c.8159C>T:p.(Thr2720Met)	VUS	maternal
PaedCan49	f	Ewing sarcoma	1.5	*SMARCA4* (AD)	het	ENST00000344626:c.3019C>A:p.(Leu1007Ile)	VUS	de novo
PaedCan50	f	Nephroblastoma	6.2	*CHEK2* (AD)	het	ENST00000328354:c.190G>A:p.(Glu64Lys)	VUS	paternal
PaedCan52	m	Nephroblastoma	1.6	*BRCA2* (AD, AR)	het	ENST00000544455:c.3515C>G:p.(Ser1172Trp)	VUS	paternal
PaedCan57	f	Alveolar rhabdomyosarcoma	14.4	*CHEK2* (AD)	het	ENST00000328354:c.85C>T:p.(Gln29*)	P	maternal
PaedCan59	m	Embryonal rhabdomyosarcoma	5.2	*MUTYH* (AR)	het	ENST00000450313.1:c.734G>A:p.(Arg245His)	P	maternal
PaedCan61	m	Alveolar rhabdomyosarcoma	3.6	*RAD51C* (AD)	het	chr17:56782798-56819433del [includes RAD51C ENST00000337432: Ex5-9]	P	paternal
PaedCan67	f	Synovial sarcoma	14.8	*BLM* (AR)	het	ENST00000355112.3:c.1642C>T:p.(Gln548*)	P	maternal
PaedCan71	m	GIST	12.5	*SDHA* (AR, AD)	het	chr5:224764-244609dup [includes SDHA ENST00000264932: Ex4-11]	LP	maternal

*AAO* age at oncological diagnosis, *NOS* not otherwise specified, *MELTUMP* Melanocytic tumours of uncertain malignant potential, *GIST* gastrointestinal stromal tumour, *FNH* focal nodular hyperplasia, *CML* chronic myeloid leukemia, *AD* autosomal-dominant, *AR* autosomal-recessive, *het* heterozygous, *hom* homozygous, *P* pathogenic variant, *VUS* variant of unknown clinical significance, *LP* likely pathogenic variant, chromosomal positions are GRCh37.

### Variants in autosomal-dominant CPS genes

Heterozygous P/LP variants in autosomal dominant (AD) CPS genes were found in seven patients (9.7%, Table 1). Of these, four patients carried a P/LP variant in an AD CPS gene known to be associated with the patient’s tumour type. A pathogenic variant in *WT1* (c.1046del, p.(Leu349Profs*26)) was found in a patient with nephroblastoma (PaedCan01). He and his brother both had nephroblastomatosis and cryptorchidism. Segregation analysis identified the variant in the brother of the index. Both parents tested negative for this variant. The combination of a negative test in the parents and a positive test in a sibling indicates gonadosomatic mosaicism, which is associated with an increased recurrence risk of a *WT1-*disorder for siblings and was suspected in this family (Table 1).

A pathogenic de novo deletion of the *NF1* gene (Ex30-58del, size 285 kb) was found in a second patient with a malignant mesenchymal tumour (PaedCan18). The patient fulfilled the clinical criteria for neurofibromatosis type 1. TGS allowed the precise detection of the genomic breakpoint locations and the surrounding genes affected by the deletion. In this case, none of the surrounding genes was known to be disease-causing in OMIM.

The third patient had a history of papillary thyroid carcinoma (PaedCan16) and was found to carry a pathogenic *PALB2* variant (c.72del, p.(Arg26Glyfs*7)) as well as a pathogenic deletion of several exons of *DICER1* (Ex1-7del, size 1.11 Mb), both inherited from her mother. Besides the diagnosis of breast cancer at the age of 49 years in the patient’s mother, there was no additional cancer diagnosis related to the germline findings in the family. However, thyroid gland diseases of the mother and the sister were reported. In addition to *DICER1*, three additional disease-causing OMIM genes for recessive conditions or conditions of unknown inheritance (*GLRX5*, *TCL1B, TCL1A*) were found to be within the deletion.

The fourth patient was diagnosed with a gastrointestinal stromal tumour (GIST) (PaedCan71), in whom we identified a variant in *SDHA*, a heterozygous duplication of exon 4-11 (Table 1), initially classified as VUS. Sequencing of the patient’s tumour tissue confirmed the duplication and indicated additional loss of the wildtype allele. Immunohistochemistry showed a loss of SDHB expression in the tumour tissue, which indicates SDH deficiency that can be associated with loss of *SDHA* as well. The duplication was inherited from the patient’s mother, who was diagnosed with malignant melanoma at the age of 56 years. However, she did not report clinical signs of hereditary paraganglioma-pheochromocytoma syndrome, like catecholamine-related symptoms such as hypertension crisis, tachycardia or dizziness. Based on the immunohistochemistry findings, suspected loss of the wild type allele in tumour tissue and the tumour type of the index, the variant was reclassified as a likely pathogenic variant.

Three patients carried a P/LP variant in an AD CPS gene not known to be associated with the patient’s specific tumour type. We found a likely pathogenic variant in *MITF* (c.952 G > A, p.(Glu318Lys)) in a patient, who was diagnosed with neuroblastoma at the age of 2 months (PaedCan02). The variant was inherited from the patient’s healthy mother. The family history was unremarkable for *MITF-*associated cancer types. In another patient with alveolar rhabdomyosarcoma (PaedCan57) a pathogenic variant in *CHEK2* (c.85 C > T, p.(Gln29*)) was found, which is currently not thought to be causative for the development of sarcoma. Diagnostic tumour testing for therapeutic decision support did not detect a loss of the second allele in tumour tissue in this patient. The variant was inherited from the patient’s healthy mother. However, the family history was unremarkable for *CHEK2*-associated malignancies. In an additional patient (PaedCan61), a deletion of *RAD51C* (Ex 5-9del, size 0.37 Mb) was found for which relation to embryonal rhabdomyosarcoma is not known. This variant was inherited from the patient’s father but his family history was unsuspicious for other *RAD51C*-associated tumours.

Besides P/LP variants, we found variants of unknown clinical significance (VUS) in five patients. In one patient (PaedCan08) we found two VUS, one in *ATM* (c.1516 G > T, p.(Gly506Cys)) and one in *PALB2* (c.1843C>G, p.(Pro615Ala)). This patient was diagnosed with large cell anaplastic lymphoma at the age of 2 years, basal cell carcinoma of the skin at the age of 17, urothelial carcinoma at the age of 25 years, and invasive ductal mammary carcinoma at the age of 27 years. The patient received chemotherapy and whole-body irradiation after initial diagnosis. The patient’s mother was diagnosed with Hodgkin-lymphoma at 35 years and breast cancer at 52 years of age. VUS were also detected in *NF1* in a patient without clinical symptoms of NF1, *BRCA2* in a patient with nephroblastoma, *SMARCA4* in a patient with Ewing sarcoma, and *SEC23B*, a candidate gene for Cowden, in a patient with multiple tumours and an additional pathogenic heterozygous *RECQL4* variant.

### Variants in autosomal-recessive CPS genes

**Fig. 2:**
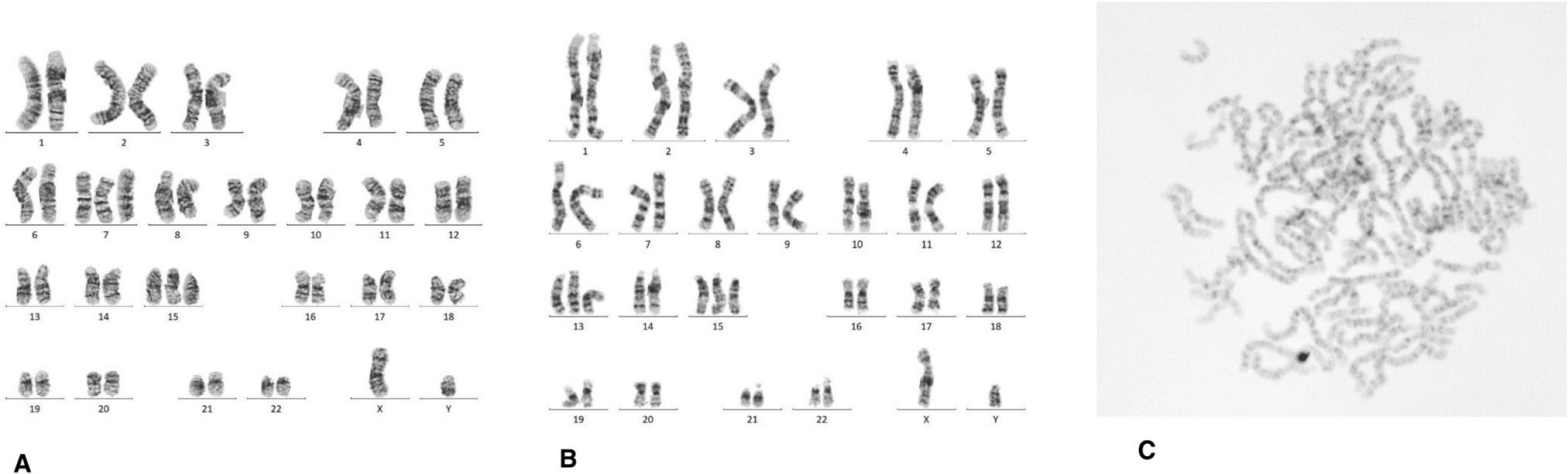
Karyogram of a patient with mosaic variegated aneuploidy. Karyotyping revealed several different numerical abnormalities in 10 of 23 analysed mitoses of patients PaedCan07. **A** Mitosis with 48,XY, + 7, + 15, **B** 48,XY, + 13, + 15. Panel **C** shows an example of a mitosis with premature chromatid separation, as it was found in about one third of the analysed cells.

P/LP variants in autosomal-recessive CPS genes were found in six patients (8.3%, Table 1). While most variants were heterozygous and therefore of unknown relevance (Table 1), one patient with embryonal rhabdomyosarcoma (PaedCan07) carried two compound-heterozygous likely pathogenic variants in *BUB1B*. A heterozygous frameshift variant (c.2405_2406del, p.(Ser802Cysfs*29)) was inherited from his healthy father, a likely pathogenic second variant in *BUB1B* (c.2577+5 G > A, p.(?)) was inherited from his healthy mother. The index presented with intrauterine growth retardation, microcephaly, speech delay, bilateral undescended testicles, a persistent foramen ovale, bilateral buckling drop feet and dysmorphic features of the face. During therapy he developed an encephalopathic ifosfamide toxicity and a chronic renal failure grade II-III with tubulopathy. Subsequent karyotyping of lymphocytes revealed complex mosaicism with several aneuploidies, including trisomies and monosomies, occurring in diverse combinations, and premature chromatid separation in about one third of the analysed mitoses (Fig. 2), confirming the functional relevance of the identified variants and a diagnosis of variable mosaic aneuploidy syndrome (OMIM #257300).

Another patient, who was diagnosed with neuroblastoma at the age of 3 years (PaedCan09), carried a heterozygous pathogenic variant in *RECQL4* (c.1573del, p.(Cys525Alafs*33)), which was inherited from her mother. The patient was diagnosed with several additional non-malignant / borderline tumours: a fibroma of the ovary and a basal cell carcinoma at the age of 20 years, a melanoacanthoma at the age of 20 years, condylomata acuminata without detection of HPV DNA, a solitary thyroid adenoma at the age of 21 years, and a focal nodular hyperplasia of the liver at 22 years. The family history revealed a ductal carcinoma in situ with transition to poorly differentiated invasive breast carcinoma in her mother at 49 years of age and further malignant diseases in family members after the age of 70 years. In this patient, an additional VUS in *SEC23B* (c.2101 C > T, p.(Arg701Cys)) was found, which was inherited from her mother. Finally, multiple occurrences of heterozygous pathogenic variants were found in *MUTYH* and a pathogenic variant in *BLM* in a patient with synovial carcinoma (Table 1).

### Complex clinical and genetic cases

In patient PaedCan02, an additional hemizygous VUS in *GPC3* (chrX:133012559-133013559del) inherited from his mother was reported. In this patient, there were no clinical signs of Simpson-Golabi-Behmel-Syndrom (SGBS) as pre- and postnatal tall stature, facial dysmorphia or other developmental abnormalities could not be detected. Although clinical data did not confirm SGBS, a role in tumour development cannot be excluded. Another patient (PaedCan43) was found to carry a de-novo VUS in *ARID1B* (c.6728 T > C, p.(Leu2243Pro)). The patient was diagnosed with Ewing sarcoma at the age of 20 years. De-novo mutations in *ARID1B* are known to be associated with Coffin-Siris syndrome type 1 (CSS1). In this case, the patient was diagnosed with an autism-spectrum disorder and intellectual disability, but lacked typical signs of CSS1. Symptoms of a syndromic condition were found but the role of the variant in tumour development remains unclear.

### Secondary findings in other genes

Clinically relevant secondary findings (P/LP variants) were found in one patient and four unrelated parents (2.3%, *n* = 216). In a male patient (PaedCan07), we detected a pathogenic variant in *G6PD* (c.982 G > C, p.Asp328His). The variant was inherited from the patient’s mother. Despite the presence of a pathogenic variant in the X-linked Glucose-6-Phosphate Dehydrogenase gene, no haemolytic crisis occurred before the diagnosis of rhabdomyosarcoma, and no excessive transfusions were necessary during chemotherapy. As a newborn, the patient did not show a severe jaundice, significantly elevated levels of bilirubin or prolonged anaemia during treatment of the tumour. Moreover, we detected a number of pathogenic variants in the parents of several index patients including a likely pathogenic *CHEK2* variant (c.349 A > G, p.(Arg117Gly)) in the mother of PaedCan55, pathogenic variants in *RYR1* (c.7272 C > T, p.(Arg2458Cys)) and *MUTYH* (c.536 A > G, p.(Tyr179Cys)) in the mother of PaedCan10, and pathogenic variants in *APOB* (c.10580 G > A, p.(Arg3527Gln)) in two fathers (PaedCan48 and PaedCan63). Notably, the parents were all healthy with no indication of a corresponding disease at the time of writing. All parents were informed about their genetic risk to develop respective diseases and clinical follow up management was offered according to the respective guidelines.

### Clinical criteria and genetic test results

**Fig. 3:**
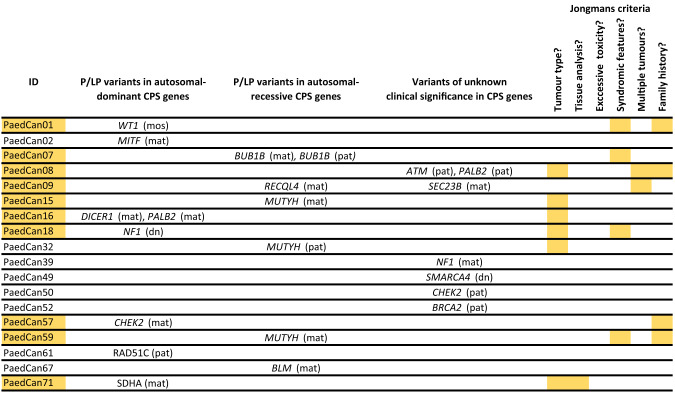
Patients with genetic findings and Jongmans criteria. Yellow squares Jongmans criterium is fulfilled, na data not available, mos germline mosaicism, mat maternal, pat paternal, dn de-novo.

Based on the modified Jongmans criteria, genetic evaluation would have been offered to 30 patients (41.6%). Of those, 18 had a tumour indicative for a CPS, seven had a family history suspicious for CPS, eight patients showed characteristic clinical features such as signs of neurofibromatosis type 1 (*n* = 2), congenital malformations (*n* = 3) or combined developmental disorders (*n* = 3, Fig. 3). Three patients presented with multiple tumours. Five patients had a combination of two different criteria and one patient was found positive for three criteria. An exceptionally high level of toxicity during treatment was not observed in our patients; the most common treatment-related toxicities included Ifosfamide-induced encephalopathy (*n* = 5) and renal impairment/renal Fanconi syndrome (*n* = 4). Family history was missing for five patients. Of all patients fulfilling the Jongmans criteria, ten were found to carry a P/LP variant (33.3%) including five patients with a P/LP variant in an autosomal-dominant CPS gene. A de novo variant and a gonadosomatic mosaic was found in one patient each (Table 1). Genetic evaluation would not have been recommended for two patients with a P/LP variant in an AD CPS gene and one patient with a heterozygous P/LP variant in a recessive gene. Those variants were not known to be associated with the patient’s specific tumour types and the role of heterozygous variants in recessive genes is unclear today.

## Discussion

In this study, we used clinical child-parent TGS in a cohort of 72 unselected patients with childhood cancer. The overall rate of P/LP variants in CPS genes was 18.1%, and 9.7% in autosomal-dominant CPS genes. Comparable studies have found similar rates between 8% and 18% in childhood cancer patients [5, 7–12, 33, 34].

In our study, four patients carried a P/LP variant in an autosomal-dominant gene associated with their tumour type and paediatric onset. The association of germline findings and childhood cancer for the other patients is currently unknown. For example, we found a LP variant in *MITF* in a child with neuroblastoma. *MITF* is a known moderate risk gene for adult-onset melanoma [35]. Two additional neuroblastoma patients with a LP *MITF* variant were reported previously by Fiala et al. (2021). Although no further functional evidence was available (i.e., loss of the second allele in tumour tissue), the identification of three LP variants in *MITF* in 194 patients in both studies combined is higher than expected from published data in an adult population (gnomAD: 242/152114, one patient homozygous). Heterozygous variants in recessive genes like *RECQL4* which is associated with Rothmund-Thomson syndrome type 2 are another example. Heterozygous *RECQL4*-variants were found to be enriched in paediatric osteosarcoma patients [36]. In our cohort, we found a heterozygous *RECQL4* variant in a neuroblastoma patient. P/LP variants in CPS genes not known to be related with the child’s tumour are a challenge in terms of clinical management of index patients and their families requiring individualized cancer prevention strategies.

TGS is not yet a standard test in diagnostic laboratories, especially since sequencing costs are still high and data analysis is complex requiring extensive bioinformatic expertise [37]. With the comprehensive nature of genome sequencing, TGS has advantages over conventional diagnostic strategies like single exome or panel sequencing and allows for example the parallel analysis of different inheritance modes [9]. In our cohort, pathogenic de novo variants were found in *NF1 a*nd *WT1*. The *WT1* variant was later categorized as suspected gonadosomatic mosaicism, a known phenomenon in *WT1* families, as both children carried the pathogenic *WT1* germline variant [38–40]. Another example is the higher sensitivity for the detection of structural variants of genome sequencing compared to panel or exome sequencing [15, 18]. Besides the relative copy number values, we were able to determine precise breakpoint locations helping to identify potential additional clinically relevant findings in neighbouring genes. And finally, the general usage of exome or genome sequencing is further supported by the detection of multiple pathogenic variants in CPS genes in a patient with papillary thyroid cancer.

TGS is a broad sequencing approach that allows the identification of secondary findings outside of CPS genes in both children and their parents. In our cohort, a secondary finding was reported in 2.3% of cases. The variants were reported to the families whenever it was of direct relevance to the carrier in accordance with the ACMG guidelines and appropriate clinical management was recommended. The detection rate of secondary findings was slightly increased compared to previous studies that reported such findings in 1-2% of cases [41–43]. This difference can most likely be attributed to the smaller sample size of this study and differences in the published gene sets.

Conventional genetic counselling and testing strategies are based on family history, tumour entity, and age at onset. Family history alone does not seem to be a sufficient marker to identify carriers of P/LP variants in CPS genes, as only 40% of cases with a P/LP variant in a CPS gene were found have a family history indicative for CPS [5]. Therefore, extended clinical criteria catalogues were developed that include additional parameters such as, the occurrence of second malignancies, specific malignancies with a high rate of underlying genetic cancer disposition, excessive toxicity, syndromic characteristics, and paediatric patients with rare or adult-type tumours [2, 3]. Based on the Jongmans criteria, genetic evaluation would have been recommended to 30 patients. All patients with a P/LP variant in a gene likely to be causative for their disease would have been identified by this questionnaire. This is in line with two previous studies reporting the identification of 100% (modified Jongmans criteria) and 80.9% (classic Jongmans criteria) of patients with P/LP variants in CPS genes [11, 44]. The study design does not allow to evaluate if all the patients would have been offered a genetic test based on the recommendation of genetic counselling, but this study confirms the high sensitivity of current clinical criteria to identify at risk patients. An unselected approach, as used here, still might prove beneficial as it is independent of a manifest disease in the parents and accounts for incomplete penetrance, gender-specific cancer risks and environmental exposure, which can reduce the informative value of family history [6]. In addition, the concern of a relevant number of parents regarding a predisposition to tumour diseases in their family supports the general offer of genetic testing.

Limitations of this study are the sample size and cohort composition, which did not include leukaemia, tumours of the central nervous system and retinoblastoma and therefore does not allow us to comment on the overall detection rate of P/LP germline variants in children with malignancies.

This study used TGS to evaluate its utility and applicability in a cohort of unselected paediatric cancer patients in a clinical setting. Overall, we show that TGS has advantages over other sequencing approaches which include the availability of immediate information on inheritance supporting variant classification as well as the characterisation of structural variants with base-pair resolution. Further, clinical TGS provides data that can be used for later re-analysis and identification of candidate genes and novel gene-disease associations [45]. TGS holds the promise to allow rapid retrospective identification of variants in future cancer genes or combinatorial effects of single genomic variants. TGS can be used as an efficient first-tier diagnostic test that, with continuously decreasing sequencing costs, will quickly enter the clinical space in childhood cancer and other conditions.

### Supplementary information

Supplemental Material

## Data Availability

The data generated in this study are available upon request from the corresponding author.
