# Supplementary material for: Clinical trio genome sequencing facilitates the interpretation of variants in cancer predisposition genes in paediatric tumour patients
Source: Eur J Hum Genet. 2023 Jul 28;31(10):1139–46. doi: 10.1038/s41431-023-01423-8 (PMC10545765; doi:10.1038/s41431-023-01423-8)
Supplement: Supplementary file 1 — Supplemental Material [file 41431_2023_1423_MOESM1_ESM.docx]

**Supplementary Table 1: Gene list of CPS genes**

Regions: 4076
Bases: 833569
Genes: 266

*ABCB11, ABRAXAS1, ACAN, ACD, AGL, AIP, AKT1, ALK, APC, ASXL1, ATG2B, ATM, ATR, AXIN2, BAP1, BARD1, BLM, BMPR1A, BRAF, BRCA1, BRCA2, BRIP1, BUB1, BUB1B, BUB3, CABLES1, CASR, CBL, CCND1, CD70, CDC73, CDH1, CDH23, CDK4, CDKN1B, CDKN1C, CDKN2A, CDKN2B, CDKN2C, CEBPA, CEP57, CHEK2, COL7A1, CREBBP, CTC1, CTNNA1, CTNNB1, CTNND1, CTR9, CTRC, CYLD, DDB2, DDX41, DICER1, DIS3L2, DKC1, DOT1L, EED, EGFR, EGLN1, EGLN2, ELANE, ELP1, EP300, EPAS1, EPCAM, ERCC1, ERCC2, ERCC3, ERCC4, ERCC5, ESR1, ETV6, EXT1, EXT2, EZH2, FAH, FAN1, FANCA, FANCB, FANCC, FANCD2, FANCE, FANCF, FANCG, FANCI, FANCL, FANCM, FBXO24, FH, FLCN, G6PC1, GALNT12, GALNT14, GATA2, GFI1, GLMN, GPC3, GPC4, GPR161, GREM1, GSKIP, HAX1, HMBS, HNF1A, HNF1B, HOXB13, HPD, HRAS, IKZF1, IL1B, IL1RN, INSR, IPMK, JAK2, KDR, KIF1B, KIT, KLHDC8B, KMT2D, KRAS, L2HGDH, LIG4, LZTR1, MAD2L2, MAP2K1, MAP2K2, MAP3K6, MAX, MC1R, MEN1, MET, MGMT, MITF, MLH1, MPL, MRE11, MSH2, MSH3, MSH6, MSR1, MTAP, MUTYH, NBN, NEK11, NF1, NF2, NHP2, NKX2-1, NOP10, NOTCH3, NPAT, NRAS, NSD1, NTHL1, NTRK1, OTC, PALB2, PALLD, PARN, PAX5, PBRM1, PDGFRA, PDGFRB, PHOX2B, PIK3CA, PMS1, PMS2, POLD1, POLE, POLH, POT1, POU6F2, PPOX, PRF1, PRKAR1A, PRKN, PRSS1, PTCH1, PTCH2, PTEN, PTPN11, PTPRT, RAD50, RAD51, RAD51C, RAD51D, RAF1, RASAL1, RB1, RECQL, RECQL4, REST, RET, RFWD3, RHBDF2, RINT1, RIT1, RNF43, RPL11, RPL19, RPL35A, RPL5, RPS10, RPS17, RPS19, RPS20, RPS24, RPS26, RPS7, RTEL1, RUNX1, SAMD9, SAMD9L, SASH1, SBDS, SCG5, SDHA, SDHAF2, SDHB, SDHC, SDHD, SEC23B, SETBP1, SH2B3, SHOC2, SLC25A11, SLX4, SMAD4, SMAD9, SMARCA4, SMARCB1, SMARCE1, SMO, SOS1, SPINK1, SPRED1, SPRTN, SRGAP1, SRP72, STK11, SUFU, TAT, TBXT, TERF2IP, TERT, TGFBR2, TINF2, TMEM127, TP53, TRIM28, TRIM37, TSC1, TSC2, UBE2T, USB1, VHL, WAS, WRAP53, WRN, WT1, XPA, XPC, XRCC2, XRCC3, YAP1*

**Supplementary Table 2: Additional Clinical information of included patients**

**AAO** - age at oncological diagnosis, **NOS** - not otherwise specified, **MELTUMP** - Melanocytic tumours of uncertain malignant potential, **GIST** - gastrointestinal stromal tumour, **FNH** - focal nodular hyperplasia, **CML** - chronic myeloid leukemia, CR – complete response

| Trio ID | Sex | AAO years | Tumours | Distant metastases at diagnosis | Relevant findings in tumour molecular profiling / pathology | Relapse (count) | Status at last follow-up |
| --- | --- | --- | --- | --- | --- | --- | --- |
| PaedCan01 | m | 0.6 | Nephroblastomatosis | no | not performed | no | CR |
| PaedCan02 | m | 0.2 | Neuroblastoma | yes | not performed | no | CR |
| PaedCan03 | f | 4.3 | Nephroblastoma | unclear | not performed | no | CR |
| PaedCan04 | f | 4.7 | Alveolar rhabdomyosarcoma | no | Translocation *FOXO1* | no | CR |
| PaedCan05 | f | 0.7 | Hepatoblastoma | no | not performed | yes (1) | CR |
| PaedCan06 | m | 14.3 | Osteosarcoma | yes | - | yes (2) | Active disease, Disease progression |
| PaedCan07 | m | 1.2 | Embryonal rhabdomyosarcoma | no | - | no | CR |
| PaedCan08 | f | 2.6  17.4  25.0  27.2 | Non-Hodgkin Lymphoma,  Basal cell carcinoma,  Urothelial carcinoma,  Breast Cancer | n/a  no  no  no | unclear | no  no  no  no | CR |
| PaedCan09 | f | 3.0  20.2  20.8  21  22 | Neuroblastoma,  fibroma of the ovary,  basal cell carcinoma,  melanocanthoma,  FNH of the liver | yes  no  no  no  no | not performed | no  no  no  no  no | CR |
| PaedCan10 | F | 5.4 | Neuroblastoma | yes | Mutation *SMARCB1* | yes (1) | DOD |
| PaedCan11 | m | 13.2 | Malignant rhabdoid tumour | no | Translocation *EWSR1* | no | CR |
| PaedCan12 | f | 4.4 | Ewing sarcoma | yes | No 1p deletion, no *MYCN* amplification | no | CR |
| PaedCan13 | f | 15.8 | Neuroblastoma | no | not performed | no | CR |
| PaedCan14 | f | 0.5 | MPNST | unclear | not performed | yes (1) | CR |
| PaedCan15 | m | 9.7 | MELTUMP | unclear | not performed | no | CR |
| PaedCan16 | f | 15.7 | Papillary thyroid cancer | no | not performed | no | CR |
| PaedCan17 | m | 13.3 | Embryonal rhabdomyosarcoma | no | - | no | CR |
| PaedCan18 | m | 12.1 | Low grade malignant mesenchymal tumour, NOS | unclear | - | yes (1) | Active disease, Disease progression |
| PaedCan19 | f | 10.5 | Dysgerminoma | no | not performed | no | CR |
| PaedCan20 | m | 14.5 | Alveolar rhabdomyosarcoma | yes | Translocation *FOXO1* | yes (1) | DOD |
| PaedCan21 | m | 4.8 | Nephroblastoma | no | not performed | no | CR |
| PaedCan22 | m | 14.5 | Ewing sarcoma | yes | Translocation *EWSR1* | yes (2) | Active disease, Partial Response |
| PaedCan23 | f | 0.7 | Malignant ectomesenchymoma | no | - | no | CR |
| PaedCan24 | f | 0.0 | Neuroblastoma | yes | No 1p deletion, no *MYCN* amplification | no | CR |
| PaedCan25 | f | 1.8 | Nephroblastoma | no | not performed | no | CR |
| PaedCan26 | f | 2.3 | Neuroblastoma | yes | 1p deletion, no *MYCN* amplification | no | CR |
| PaedCan27 | m | 8.0 | Burkitt lymphoma | n/a | Rearrangement *c-MYC* | no | CR |
| PaedCan28 | f | 15.4 | Myoepithelial salivary gland carcinoma | no | not performed | no | CR |
| PaedCan29 | m | 11.4 | Alveolar rhabdomyosarcoma | yes | Translocation *FOXO1* | yes (1) | Active disease, Disease progression |
| PaedCan30 | m | 6.4 | Nephroblastoma | yes | not performed | no | CR |
| PaedCan31 | f | 9.9 | Ganglioneuroblastoma | yes | Mutation *ALK*; no 1p deletion, no *MYCN* amplification | yes (2) | Active disease |
| PaedCan32 | f | 2.6 | Inflammatory myofibroblastic tumour | no | Gene fusion *ETV6-NTRK3* | no | CR |
| PaedCan33 | m | 6.3 | Embryonal rhabdomyosarcoma | no | - | no | CR |
| PaedCan34 | m | 3.6 | Neuroblastoma | yes | Amplification *ALK*, no *MYCN* amplification | yes (1) | CR |
| PaedCan35 | m | 2.3 | Neuroblastoma | yes | Imbalance 1p, *MYCN* amplification | yes (1) | CR |
| PaedCan36 | m | 16.2 | Salivary gland carcinoma | no | not performed | no | CR |
| PaedCan37 | f | 0.9 | Plexiform neurofibroma | no | unclear | no | Active disease, Partial Response |
| PaedCan38 | m | 2.9 | Nephroblastoma | no | not performed | no | CR |
| PaedCan39 | f | 6.8 | Embryonal rhabdomyosarcoma | no | not performed | no | CR |
| PaedCan40 | f | 3.6 | Neuroblastoma | yes | Amplification *MYCN*, no 1p deletion | no | CR |
| PaedCan41 | m | 15.8 | Pleomorphic sarcoma | no | - | no | CR |
| PaedCan42 | m | 4.0 | Nephroblastoma | no | not performed | yes (1) | DOD |
| PaedCan43 | m | 20.5 | Ewing sarcoma | no | Translocation *EWSR1* | no | CR |
| PaedCan44 | m | 8.7 | Osteosarcoma | yes | not performed | no | CR |
| PaedCan45 | m | 10.9 | Ewing sarcoma | yes | Translocation *EWSR1* | no | CR |
| PaedCan46 | m | 3.2 | Neuroblastoma | yes | Imbalance 1p, no *MYCN* amplification | no | CR |
| PaedCan47 | f | 15.1 | Mixed malignant germ cell tumour | yes | not performed | no | CR |
| PaedCan48 | m | 1.8 | Neuroblastoma | yes | Imbalance 1p, amplification *MYCN*, amplification *ALK* | no | CR |
| PaedCan49 | f | 1.5 | Ewing sarcoma | no | Translocation *EWSR1* | no | CR |
| PaedCan50 | f | 6.2 | Nephroblastoma | yes | not performed | yes (1) | Active disease |
| PaedCan51 | f | 14.4 | Sertoli-Leydig cell tumour | no | not performed | no | CR |
| PaedCan52 | m | 1.6 | Nephroblastoma | no | not performed | no | CR |
| PaedCan53 | m | 0.4 | Embryonal rhabdomyosarcoma | unclear | - | yes (1) | CR |
| PaedCan54 | m | 1.6 | Neuroblastoma | yes | Deletion 1p, amplification *MYCN* | no | Lost to follow up |
| PaedCan55 | m | 12.4 | Osteosarcoma | no | - | yes (2) | DOD |
| PaedCan56 | m | 0.7 | Hepatoblastoma | unclear | unclear | no | CR |
| PaedCan57 | f | 14.4 | Alveolar rhabdomyosarcoma | yes | Translocation *FOXO1*, deletion *PTEN* | yes (1) | DOD |
| PaedCan58 | f | 6.0 | Neuroblastoma | yes | No 1p deletion, no *MYCN* amplification | yes (4) | Active disease, Disease progression |
| PaedCan59 | m | 5.2 | Embryonal rhabdomyosarcoma | no | not performed | no | CR |
| PaedCan60 | f | 2.3 | Ewing sarcoma | no | Translocation *EWSR1* | no | CR |
| PaedCan61 | m | 3.6 | Alveolar rhabdomyosarcoma | no | Fusion *MAML2-PAX3* | no | CR |
| PaedCan62 | f | 1.3 | Embryonal rhabdomyosarcoma | no | not performed | no | CR |
| PaedCan63 | m | 0.8 | Neuroblastoma | yes | Deletion 1p, amplification *MYCN* | no | CR |
| PaedCan64 | m | 3.8 | Clear cell sarcoma | yes | not performed | no | Active disease |
| PaedCan65 | f | 9.8 | Hodgkin lymphoma | yes | not performed | no | CR |
| PaedCan66 | f | 15.4 | Papillary thyroid cancer | no | not performed | no | CR |
| PaedCan67 | f | 14.8 | Synovial sarcoma | no | Translocation *SYT* | no | CR |
| PaedCan68 | m | 2.5 | Ewing sarcoma | yes | Translocation *EWSR1* | no | CR |
| PaedCan69 | m | 0.1 | Yolk sac tumour | no | not performed | no | CR |
| PaedCan70 | m | unknown | Neuroblastoma | unclear | unclear | no | unclear |
| PaedCan71 | m | 12.5 | GIST | no | Loss of SDHB immunoexpression suggesting SDH inactivation | no | CR |
| PaedCan72 | f | unknown | Extra-abdominal desmoid fibromatosis | unclear | not performed | no | Active disease |
